# Severe Bilateral Ear Pain

**DOI:** 10.5811/cpcem.2017.7.34654

**Published:** 2017-10-06

**Authors:** Curt Canine, Lawrence Masullo

**Affiliations:** Carl R Darnall Army Medical Center, Department of Emergency Medicine, Fort Hood, Texas

## CASE PRESENTATION

**Image 1:**
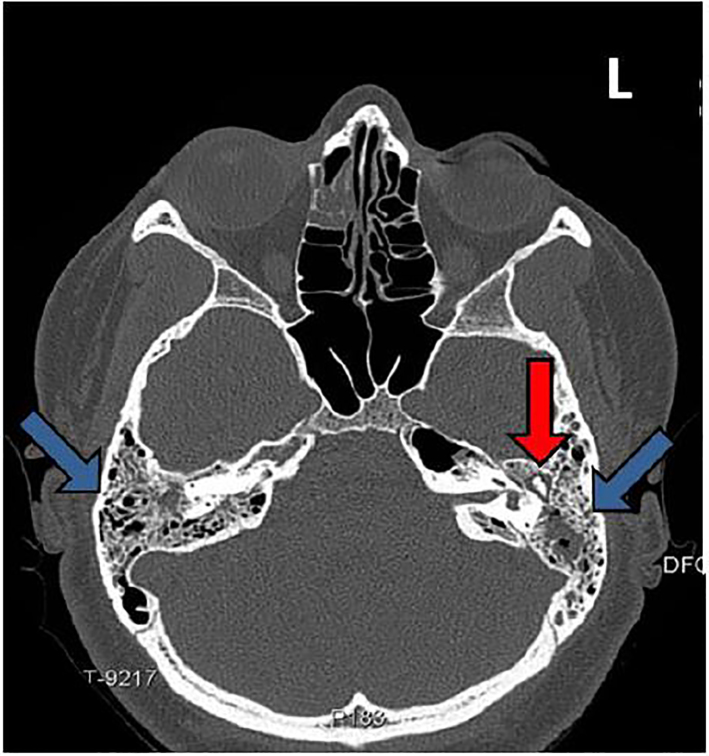
Axial computed tomography of the mastoid shows near complete opacifications of bilateral mastoid air cells with coalescence and erosions (blue arrows). Fluid is seen surrounding the ossicles of the left middle ear (red arrow).

**Image 2:**
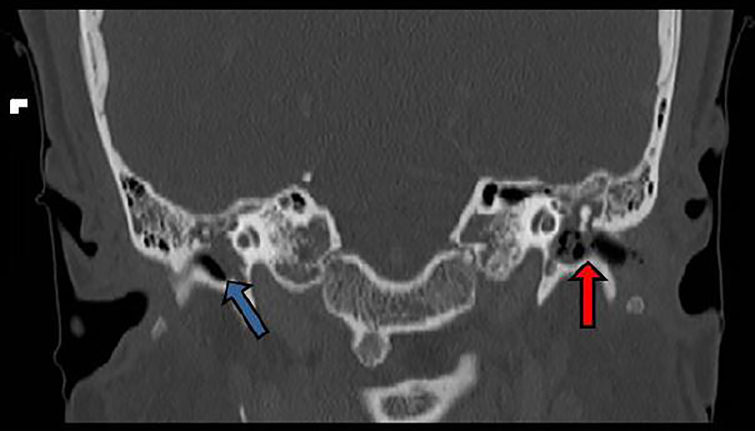
Coronal computed tomography of the mastoid demonstrates an intact right tympanic membrane with fluid in the middle ear (blue arrow). The left external auditory canal is filled with bubbly fluid, which communicated directly with the left middle ear due to a ruptured tympanic membrane (red arrow).

A 23-year-old female presented to the emergency department five weeks post-partum for headache, severe bilateral ear pain, and left ear drainage. Seven days prior she had been diagnosed with left otitis externa. Despite ofloxacin otic drops, pain progressed to involve both ears and became exacerbated by mastication or head rotation. Physical exam revealed right tympanic membrane (TM) erythema and bulging with purulent effusion; left TM completely obscured by swelling of the external auditory canal with seropurulent drainage present; moderate tenderness overlying bilateral mastoid processes; and severe pain on movement of the left ear helix. Leukocyte count (15.3 10^9^/L; 81% neutrophils), and computed tomography (CT) were obtained (Images 1 and 2).

## DIAGNOSIS

### Acute bilateral mastoiditis

Acute mastoiditis (AM) is a rare but dangerous complication of otitis media (OM) with incidence of less than one per year per 100,000, primarily affecting children (median age 2.5 years)^1^,^2^,^3^,^4^,^5^ Bilateral AM is uncommon in children (0.3% of all AM cases), but has not been reported in healthy adults. Neither has postpartum AM been described. No CT images of AM in adults have been published. The aditus ad antrum is an anatomic connection between the middle ear and the mastoid antrum. Blockage, typically a result of swelling, traps infectious material in the antrum and prevents re-aeration. Causative organisms are similar to acute OM, including *Streptococcus pneumoniae* (most common), Group A streptococcus, *Staphylococcus aureus*, and *Haemophilus influenza*.^2^,^3^,^4^,^6^

The mastoid air cells are in close proximity to the posterior cranial fossa, lateral sinuses, facial nerve canal, semicircular canals, and the petrous tip of the temporal bone. Erosions and coalescence of air cells can cause temporal lobe abscess, lateral sinus septic thrombosis, facial nerve palsy, or meningitis.^7^,^8^,^9^ Ceftriaxone and vancomycin were administered intravenously to the patient, and she was admitted to medicine. Ear, nose and throat consult recommended amoxicillin clavulanate and ciprofloxacin/dexamethasone ear drops. Symptoms and exam were improved at follow-up four days later.

**CPC-EM Capsule**

What do we already know about this clinical entity?

Mastoiditis is uncommon, affecting mostly children. Bilateral disease has not been reported in healthy adults, but may cause abscess or meningitis.

What is the major impact of the image?

Emergency clinicians should recognize historical and physical exam features of mastoiditis and have an appreciation of radiographic findings to expedite definitive treatment of the patient.

How might this improve emergency medicine practice?

The images serve as a primer for recognition of mastoiditis, and remind the emergency provider that otitis externa is not always a benign process, but may progress to more serious disease.
